# Quercetin Ameliorates Renal Injury and Pyroptosis in Lupus Nephritis through Inhibiting IL-33/ST2 Pathway In Vitro and In Vivo

**DOI:** 10.3390/antiox11112238

**Published:** 2022-11-13

**Authors:** Hsin-Yuan Chen, Yi-Fen Chiang, Yong-Han Hong, Tzong-Ming Shieh, Tsui-Chin Huang, Mohamed Ali, Hsin-Yi Chang, Kai-Lee Wang, Shih-Min Hsia

**Affiliations:** 1 School of Nutrition and Health Sciences, College of Nutrition, Taipei Medical University, Taipei 110301, Taiwan; 2 Graduate Programs of Nutrition Science, School of Life Science, National Taiwan Normal University, Taipei 106209, Taiwan; 3 School of Dentistry, College of Dentistry, China Medical University, Taichung 40402, Taiwan; 4 Graduate Institute of Cancer Biology and Drug Discovery, College of Medical Science and Technology, Taipei Medical University, Taipei 11031, Taiwan; 5 Clinical Pharmacy Department, Faculty of Pharmacy, Ain Shams University, Cairo 11566, Egypt; 6 Graduate Institute of Medical Science, National Defense Medical Center, Taipei 114, Taiwan; 7 Department of Nursing, Ching Kuo Institute of Management and Health, Keelung 20301, Taiwan; 8 School of Food and Safety, Taipei Medical University, Taipei 110301, Taiwan; 9 Nutrition Research Center, Taipei Medical University Hospital, Taipei 110301, Taiwan; 10 Graduate Institute of Metabolism and Obesity Sciences, College of Nutrition, Taipei Medical University, Taipei 11031, Taiwan; 11 TMU Research Center for Digestive Medicine, Taipei Medical University, Taipei 110301, Taiwan

**Keywords:** systemic lupus erythematosus, lupus nephritis, renal fibrosis, inflammation, interleukin-33, quercetin

## Abstract

Lupus nephritis (LN) is a common and serious symptom in patients with systemic lupus erythematosus (SLE). Tubular interstitial fibrosis is a common underlying mechanism in the development of lupus nephritis to end-stage renal failure (ESRD). Quercetin is widely proven to prevent tissue fibrosis. Therefore, the purpose of this study is to investigate the beneficial effects of quercetin on the inhibition of fibrosis and inflammation pathways in in vitro and in vivo lupus nephritis models. In the current study, MRL/lpr mice as animal models, and HK-2 human renal tubular epithelial cells were stimulated by interleukin-33 (IL-33) to mimic the cellular model of lupus nephritis. Immunohistochemical staining, immunoblotting assay, immunofluorescence staining, and quantitative real-time polymerase chain reaction assay were used. The in vivo results showed that quercetin improved the renal function and inhibited both fibrosis- and inflammation-related markers in MRL/lpr mice animal models. The in vitro results indicated that quercetin ameliorated the accumulation of fibrosis- and inflammation-related proteins in IL-33-induced HK-2 cells and improved renal cell pyroptosis via the IL33/ST2 pathway. Overall, quercetin can improve LN-related renal fibrosis and inflammation, which may offer an effective potential therapeutic strategy for lupus nephritis.

## 1. Introduction

Systemic lupus erythematosus (SLE) is the prototype systemic autoimmune disease, characterized by chronic and aberrant immune activation against nuclear self-antigens and drives the production of pathogenic autoantibodies and activation of immune complexes (ICs) that mediate organ and tissue damage, especially in the skin and kidneys [1]. Lupus nephritis (LN) is the most serious manifestation in patients with SLE, and about 35–60% of SLE patients will suffer from severe loss of renal function due to the complication of LN [2]. To date, 5–20% of LN patients still develop end-stage renal disease (ESRD) within ten years unless prevented by effective therapies [3,4]. To alleviate the worsening of the pathological process of LN, many clinical guidelines recommend reducing proteinuria and improving pathological features, such as glomerulosclerosis and tubulointerstitial fibrosis [5,6]. Tubulointerstitial fibrosis is a common pathology involved in the process of lupus nephritis development to ESRD [7], and its pathogenesis usually occurs through the secretion of pro-inflammatory factors from renal tubular epithelial cells (TECs) to recruit and activate different inflammatory cells, which, in turn, produce cytokines that drive the epithelial–mesenchymal transition (EMT) of TECs [8]. TECs finally obtain a myofibroblast phenotype, which is characterized by gain of mesenchymal features, including expression of fibronectin and vimentin, as well as collagen synthesis [8].

Interleukin-33 (IL-33), is abundant in endothelial cells, epithelial cells, and fibroblastic stromal cells [9] and is recognized as a dual function protein, acting both extracellularly as an IL-1 family cytokine, and intracellularly as a nuclear factor regulating gene expression, thus, playing a crucial role in regulating innate and adaptive immune systems [10,11]. IL-33 has been identified as a tissue-derived alarmin that is released from the nucleus of producing cells upon tissue damage or necrotic cell death following exposure to allergens or viral infection. When local immune cells receive warning signals and get activated, full-length IL-33 is processed by neutrophil-derived proteases into mature IL-33 with 10- to 30-fold higher biological activity [12]. IL-33 involves various cell signaling pathways, primarily signaling through transmembrane-form suppression of tumorigenicity-2 (ST2L, also called ST2 receptor) and forming a heterodimer with the IL-1 receptor accessory protein (IL-1RAcP) [13], resulting in the dimerization of the TIR domain [14] and eliciting the recruitment of myeloid differentiation primary response protein 88 (MyD88), IL-1R-associated kinase (IRAK), and tumor necrosis factor receptor-associated factor 6 (TRAF6), then activates the downstream mitogen-activated protein kinases (c-Jun N-terminal kinases (JNK), p38, and extracellular signal-regulated kinase (ERK)), and nuclear factor-kappa B (NF-κB) signaling pathways, which ultimately induce the inflammation response [15].

Several previous studies have suggested that IL-33 contributes to the pathogenesis of autoimmune diseases [16], especially in patients with SLE and lupus nephritis; the expression of IL-33 and its receptor ST2 is significantly upregulated [17]. In addition, serum IL-33 levels in SLE patients were significantly higher than those in healthy subjects, which are thought to be closely related to IgA, erythrocyte sedimentation rate (ESR), and C-reactive protein (CRP), speculating that IL-33 may be part of the acute phase response, even if it is not related to SLE activity [18]. Therefore, IL-33 inhibition may hinder the progression of SLE [19].

Quercetin (2-(3,4-dihydroxyphenyl)-3,5,7-trihydroxy-4Hchromen-4-one) is a bioactive natural compound that is widely found in natural plants, such as flowers, bark, and fruits and vegetables [20]. Quercetin has been widely used in various diseases due to its extensive pharmacological effects, including anti-oxidant [21], anti-inflammation [22,23], immune-modulation [24], anti-microorganism [25], and so on. Based on previous literature on kidney disease, quercetin has been reported to protect against renal damage in diabetic nephropathy (DN) animal models through anti-oxidant and anti-inflammatory effects [26]. In addition, quercetin has been reported to restore renal function in mice with renal fibrosis induced by unilateral ureteric obstruction (UUO) [27]. In the current study, we also validated the ameliorating effect of quercetin on renal injury caused by UUO (Supplementary Figure S1). Furthermore, quercetin has been reported to effectively ameliorate the kidney damage in pristane-induced lupus nephritis animal models through anti-inflammation effects [28].

Although the anti-fibrotic and anti-inflammatory properties of quercetin are well known, the underlying molecular mechanisms of quercetin potential beneficial effects on lupus nephritis remain poorly understood, in particular, the effect of quercetin on IL-33-induced lupus nephritis patterns have not yet been reported. In the current study, we aimed to investigate whether quercetin has anti-fibrosis and anti-inflammation effects on both MRL/prl mice in vivo and IL-33-induced lupus nephritis in vitro cell models, and we also aim to elucidate the underlying mechanism, which might contribute to better utilization of quercetin for lupus nephritis therapy.

## 2. Materials and Methods

### 2.1. Reagent Preparation

The yellow powder quercetin was purchased from Sigma-Aldrich (St. Louis, MI, USA) (catalog# 6151-25-3) and stored as a stock solution with a concentration of 100 mM in dimethyl sulfoxide (DMSO). Recombinant human IL-33 (carrier-free) was purchased from Biolegend (San Diego, CA, USA) (catalog# 581802) and stored as a stock solution at a concentration of 200 μg/mL in 0.1% bovine serum albumin (BSA). During the in vitro experiment, each stock solution was diluted to the desired final concentration in the medium, while DMSO and/or 0.1% BSA served as a vehicle control.

### 2.2. MRL/lpr Mice

MRL/lpr mice (characterized by lpr mutations associated with the Fas gene) are a model of spontaneous SLE that develop lupus with human-like symptoms, such as erythema and nephritis with proteinuria [29,30,31]. The 8-week-old MRL/lpr female mice were a generous gift from Dr. Yong-Han, Hong (Department of Nutrition, I-SHOU University, Kaohsiung City, Taiwan). All mice were divided into two groups: (1) SLE group: MRL/lpr mice (*n* = 5); (2) Quercetin group: MRL/lpr mice were given 40 mg/kg quercetin by gavage daily (*n* = 5). Mice were gavaged with quercetin dissolved in sterile water in a suspension form after shaking while the control group was also gavaged with sterile water every day. The body weight was recorded, and the state of the mice was observed every week. The whole experimental period was three months. At the end of the experiment, the mice were euthanized with mixture solution (1 mL Zoletil and 0.1 mL Rompun in 3.9 mL normal saline), and brain, spleen, kidneys, and uterine weights were recorded, as well as blood being collected. Harvested samples were used for analyzing the expression of specific proteins mainly in the kidneys. All animal studies were conducted according to the protocols approved by the Institutional Animal Care and Use Committee (IACUC) of Taipei Medical University (LAC-2016-0215).

### 2.3. Renal Function Assessment

Renal function was assessed through the measurement of the kidney index, blood urea nitrogen (BUN), and plasma creatinine (Cr) concentration [32]. The kidney index (mg/g) was calculated as the ratio of the weight of the two kidneys to the body weight of the mice. BUN and Cr were determined by the Department of Medical Laboratory (Taipei Medical University, Taipei, Taiwan).

### 2.4. Immunohistochemical Staining

Kidney tissue specimens were fixed using 4% paraformaldehyde and embedded in paraffin, and the subsequent staining was entrusted to BIO-CHECK LABORATORIES LTD (New Taipei City, Taiwan) for processing. Kidney sections were stained with Hematoxylin and Eosin (H&E) stain, Sirius Red stain, and Toluidine Blue stain, as well as different antibodies IL-33 (1:400, Affinity Biosciences, Cincinnati, OH, USA), NLRP3 (1:400, Novus Biologicals, Centennial, CO, USA), and IL-1β (1:400, Abcam, Cambridge, UK), and the expression changes were recorded using an EVOS^®^ FL Auto Imaging System (Thermo Fisher Scientific, Waltham, MA, USA). The determination of tubulointerstitial injury, degree of interstitial collagen deposition, and tubulointerstitial infiltration was based on previous literature [28,33,34].

### 2.5. Cell Culture and Cell Viability

HK-2 (human kidney 2, human renal tubule epithelial cell line) was purchased from Bioresource Collection and Research Center (BCRC, Hsinchu, Taiwan) and grown in Dulbecco’s Modified Eagle Medium/Ham’s F-12 Medium in a 1:1 ratio (CAISSON Labs, Smithfield, UT, USA), which was supplemented with 10% fetal bovine serum (FBS; GIBCO, Grand Island, NY, USA), 100 U/mL penicillin, and streptomycin in a humidified incubator at 37 °C with 5% CO_2_.

### 2.6. Immunofluorescence Staining

HK-2 cells (5 × 10^4^) were seeded in a 6-well microplate with glass slides (18 × 18 mm). After the cells were adhered stably, IL-33 and quercetin were added into the cells, as explained in figure legends. HK-2 cells were fixed in 4% paraformaldehyde (Sigma-Aldrich) for 10 min, then permeabilized with 0.25% Triton X-100 for 10 min and blocked by 5% bovine serum albumin (BSA) for 30 min. Adherent cells were washed twice with PBS at room temperature (RT) between each step on a shaker. HK-2 cells were then incubated with anti-vimentin (1:200, GeneTex, Irvine, CA, USA), anti-fibronectin (1:200, Abcam, Cambridge, UK), anti-NLRP3 (1:200, Novus Biologicals, Centennial, CO, USA), anti-ASC (1:200, Santa Cruz Biotechnology, Dallas, TX, USA), or anti-IL-1β (1:200, Abcam, Cambridge, UK) diluted in 5% BSA overnight at 4 °C, followed by incubation with Alexa Fluor 448-goat anti-rabbit immunoglobulin or Alexa Fluor 546-goat anti-mouse immunoglobulin antibodies (Thermo Fisher Scientific, Waltham, MA, USA) for 45 min at RT. Finally, the coverslips were mounted on the glass slides with ProLong^®^ Gold Antifade Mountant (Thermo Fisher Scientific, Waltham, MA, USA) solution (containing DAPI), sealed with nail polish, and then stored at −20 °C. Fluorescent images were recorded and photographed with EVOS^®^ FL Auto Imaging System (Thermo Fisher Scientific, Waltham, MA, USA).

### 2.7. Cytoplasmic and Nucleus Extraction Preparation

Nuclear and cytoplasmic proteins were isolated with nuclear and cytoplasmic extraction reagents, according to the manufacturer’s protocol (Nuclear/Cytosol Fractionation Kit, BioVision, Waltham, MA, USA), and the expression levels of anti-NF-κB (Cell Signaling Technology, Beverley, MA, USA) in the cytoplasmic or nuclear fractions were determined, respectively. Anti-α-tubulin (Abcam, Cambridge, UK) and anti-histone H3 (Abcam) were used as housekeeping genes to determine cytoplasmic and nuclear protein extraction purity.

### 2.8. Western Blotting Analysis

The extraction procedure for total protein was the same as in our previous study [35]. Briefly, HK-2 cells treated with a combination of IL-33 and quercetin were extracted with ice-cold lysis buffer containing protease (Roche, Basel, Switzerland) and/or phosphatase inhibitor cocktail (Roche). Cell lysates were quantified at 562 nm using an ELISA reader (BioTek, Winooski, VT, USA) after reaction with T-Pro BCA Protein Assay Kit (OMICS BIOTECHNOLOGY CO., LTD., New Taipei City, Taiwan). The proteins were mixed with SDS-PAGE loading buffer and boiled for 5 min before being subjected to SDS-PAGE gels, followed by protein transfer to a PVDF membrane. After blocking non-binding sites with 5% BSA (BioShop, Burlington, ON, Canada) for 1 h at RT, the membrane was sequentially incubated with primary antibodies of interest (at 4 °C overnight) and the corresponding goat anti-rabbit/mouse secondary antibody IgG (Abcam, Cambridge, UK) (for 1 h at RT). Membranes were washed three times with Tris-buffered saline buffer with Tween-20 (TBST, OMICS BIOTECHNOLOGY CO., LTD.) for 30 min (60 rpm) before changing each buffer. After reacting with electrochemiluminescence (ECL; Thermo Fisher Scientific) in the dark, imaging was performed with an eBlot Touch ImagerTM (eBlot Photoelectric Technology, Shanghai, China). Densitometric estimations were quantified using the Image J software (National Institutes of Health, Bethesda, MD, USA). The antibody details were provided as follows: anti-IL-33 (Affinity Biosciences, Cincinnati, OH, US), anti-GSDMD (Affinity Biosciences), anti-NLRP3 (Novus Biologicals, Centennial, CO, USA), anti-IL-6 (Novus Biologicals), anti-HMGB1 (Novus Biologicals), anti-vimentin (GeneTex, Irvine, CA, USA), anti-TLR9 (GeneTex), anti-IL1RAP (GeneTex), anti-ASC (Santa Cruz Biotechnology, Dallas, TX, USA), anti-caspase-1 (Santa Cruz Biotechnology), anti-TLR4 (Santa Cruz Biotechnology), anti-IκBα (Cell Signaling Technology, Beverley, MA, USA), anti-MyD88 (Cell Signaling Technology), anti-phospho-AMPKα (Cell Signaling Technology), anti-fibronectin (Abcam, Cambridge, UK), anti-IL-1β (Abcam), anti-IL-8 (Proteintech, Rosemont, IL, USA), anti-ST2 (Proteintech), anti-TLR7 (Proteintech), and anti-horseradish peroxidase (HRP)-conjugated glyceraldehyde 3-phosphate dehydrogenase (GAPDH; Proteintech). The dilution ratios of the antibodies used are detailed in Supplementary Table S1.

### 2.9. RNA Extraction and Real-Time Quantitative PCR (RT-qPCR)

Procedures of total RNA isolation, reverse transcription, and qPCR were modified from our previous study [35]. In brief, total RNA from HK-2 cells treated with a combination of IL-33 and quercetin-treated was extracted using TRIzol™ Reagent (Invitrogen, Carlsbad, CA, USA) followed by purification with Direct-zol RNA MiniPrep (Thermo Fisher Scientific, Waltham, MA, USA), and samples were quantified at 260 and 280 nm using an ELISA reader (BioTek, Winooski, VT, USA). The cDNA was reverse transcribed from 2 µg of total RNA using the PrimeScript^®^ RT Kit (Takara, Kusatsu, Shiga, Japan), followed by mixing with gene-specific primers and Power SYBR Green Master Mix (Thermo Fisher Scientific, Waltham, MA, USA). The mRNA level of the gene of interest was analyzed using the Applied Biosystems StepOnePlus™ Real-Time PCR System (Thermo Fisher Scientific, Waltham, MA, USA), and the amplification conditions were: denaturation at 95 °C for 10 min followed by 40 cycles for 15 s at 95 °C and 30 s at 60 °C. Synthesis of DNA product of the expected size was confirmed by DNA electrophoresis and melting curve analysis. All reactions were performed in triplicate, and relative gene expression was calculated by normalization to *GAPDH* and calculated by 2^−∆∆Ct^ method. The primer sequences used are detailed in Supplementary Table S2.

### 2.10. Statistical Analysis

All data were presented as the mean ± standard deviation (SD). For comparison of two groups, statistical differences between the means were analyzed by Student’s *t*-test using the SigmaPlot, version 12.5 (SoftHome, Taipei, Taiwan). The difference between two means was considered statistically significant when *p* < 0.05 and highly significant when *p* < 0.001.

## 3. Results

### 3.1. Quercetin Relieves Symptoms of Lupus Nephritis in MRL/lpr Mice Model

**Figure 1:**
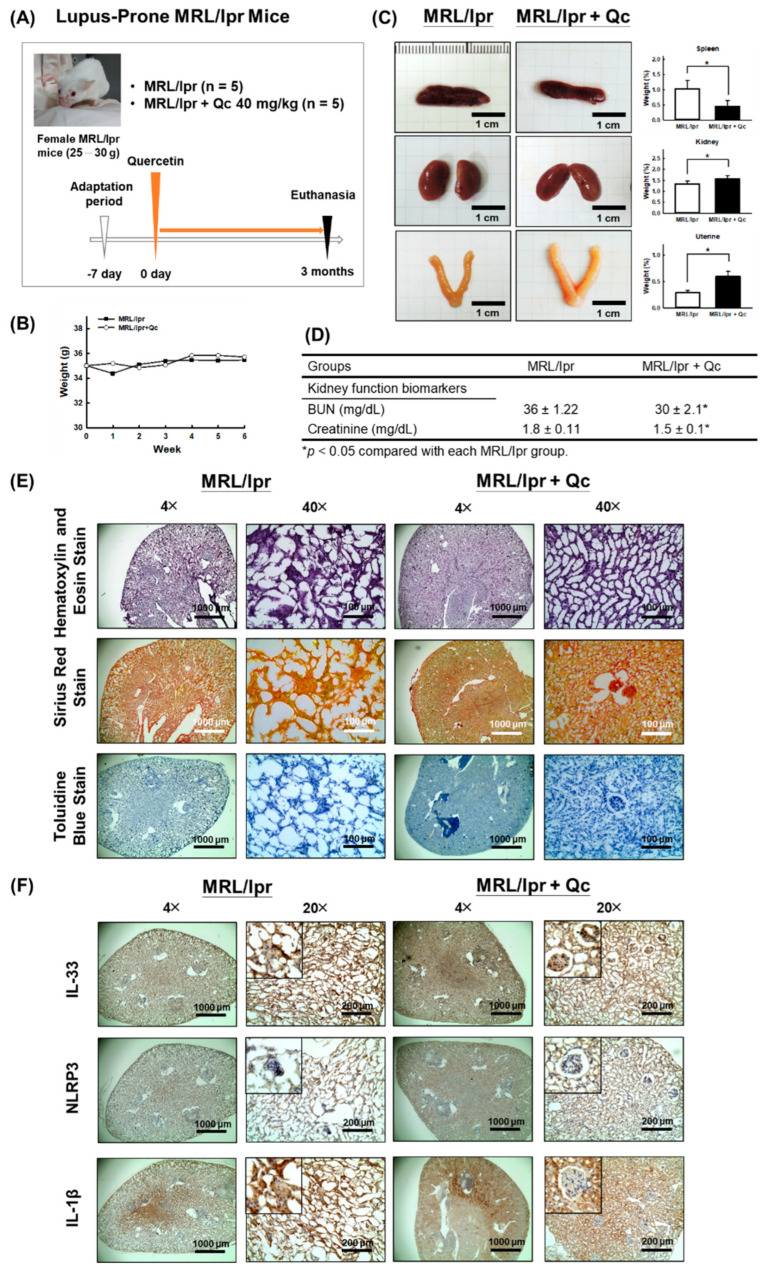
Quercetin ameliorated the renal pathology in MRL/lpr mice. (**A**) In vivo experimental procedure. (**B**) Body weight change in MRL/lpr mice. (**C**) Changes in appearance and weight of spleen, kidney, and uterus in MRL/lpr mice. (**D**) Determination of BUN and plasma creatinine concentration. (**E**) Renal tissue specimen stained with hematoxylin and eosin stain, Sirius Red stain, or Toluidine Blue stain for MRL/lpr mice that were given either vehicle (ddH_2_O) or quercetin for 3 months. Magnification, 40× and 400×. (**F**) Representative photomicrographs of the expression and distribution of IL-33, NLRP3, and IL-1β in renal tissues in MRL/lpr mice using immunohistochemical staining. Magnification, 40× and 200×. Data were expressed as mean ± SD for each group. * *p* < 0.05 versus untreated MRL/lpr mice (*n* = 5).

MRL/lpr mice are often used as an animal model to study lupus erythematosus nephritis [31]; the experimental process is shown in Figure 1A. The results showed that the body weight of MRL/lpr mice did not change significantly during the whole experimental period (Figure 1B), while it was found that daily treatment with 40 mg/kg quercetin induced hyperactivity, but there was no significant difference in the weight and appearance of the brains among the groups (results not shown). The spleen is a crucial organ in the occurrence of immune inflammation. Our results found that the spleen weight of MRL/lpr mice in the quercetin group was significantly lower than that in the untreated group (Figure 1C), indicating that quercetin reduced the enlargement of the spleen and improved the abnormal immune function or the inflammatory status of MRL/lpr mice. In addition, our results showed that the kidneys of treated MRL/lpr mice were heavier than the untreated group, which may be related to the improvement of renal atrophy (Figure 1C). Furthermore, quercetin significantly reduced the BUN and creatinine concentration of treated MRL/lpr mice, compared with the untreated group (Figure 1D).

### 3.2. Quercetin Attenuates the Pathological Changes in MRL/lpr Mice Model

To confirm that quercetin has a protective effect on the kidney tissue of MRL/lpr mice, immunohistostaining was used. The morphological alterations were evaluated by H&E staining, collagen deposition in the tubulointerstitium was assessed by Sirius Red staining, as well as mast cell infiltration into the tubulointerstitium being measured by Toluidine Blue staining. As shown in Figure 1E, the structure of the kidney tissue was scattered in the untreated MRL/lpr group, while daily administration of 40 mg/kg quercetin improved renal integrity and the level of collagen deposition (Figure 1E). However, the differences in the degree of staining after quantification did not confirm the improvement of tubulointerstitial infiltration by quercetin due to the excessively dispersed renal tissue architecture in the untreated MRL/lpr mice group.

In addition, we measured inflammation-related indicators in kidney tissue of MRL/lpr mice using immunohistochemical staining and found that quercetin showed a tendency to reduce the distribution of positive staining indicators IL-33, NLRP3, and IL-1β (Figure 1F). Summarizing the results of animal experiments, we speculate that quercetin has potential benefits in ameliorating the symptoms and progression of lupus nephritis.

### 3.3. Quercetin Reduces IL-33-Induced Fibrosis in Human Renal Tubular Epithelial Cell Line

**Figure 2:**
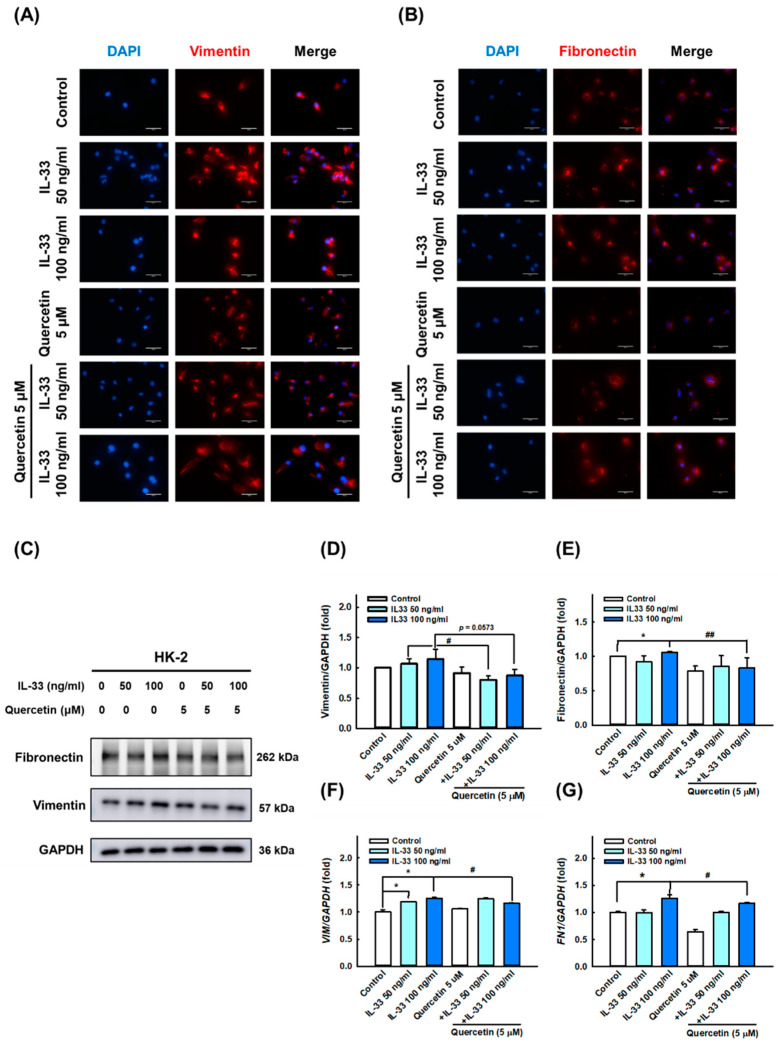
Quercetin reduced fibrosis-related proteins expression in human renal tubular epithelial cell line. HK-2 cells were co-cultured with 5 μM quercetin and 50 ng/mL or 100 ng/mL recombinant human IL-33 for 48 h. (**A**,**B**) Representative photomicrographs show the immunofluorescence staining for vimentin and fibronectin among different groups. Magnification, 400×. (**C**–**E**) Immunoblots and quantitative histograms showing the results of Western blotting analysis for the expression of vimentin and fibronectin protein. (**F**–**G**) Quantitative histograms showing the results of qPCR analysis for *VIM* and *FN1* mRNA level. Data are expressed as mean ± SD (*n* = 3). * *p* < 0.05 versus control group; ^#^ *p* < 0.05 versus corresponding IL-33-stimulated group; ^##^ *p* < 0.001 versus corresponding IL-33-stimulated group.

**Figure 3:**
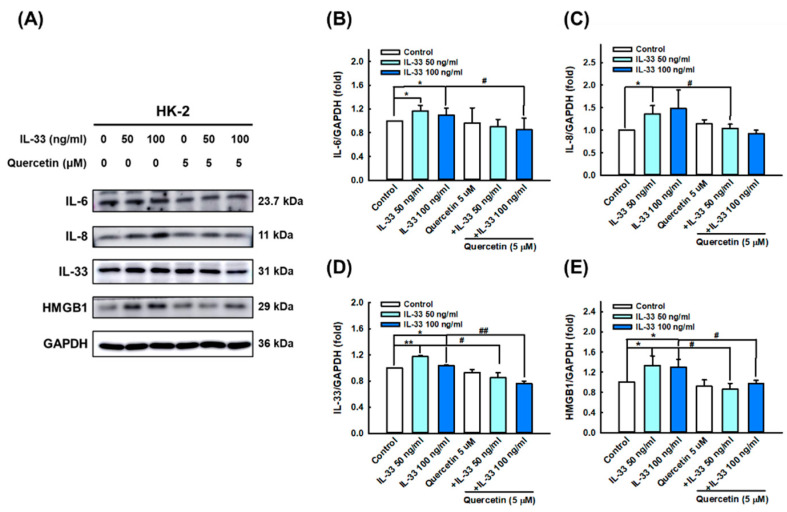
Quercetin diminished inflammatory cytokines expression in human renal tubular epithelial cell line. HK-2 cells were co-cultured with 5 μM quercetin and 50 ng/mL or 100 ng/mL recombinant human IL-33 for 48 h. (**A**–**E**) Immunoblots and quantitative histograms showing the results of Western blotting analysis for the expression of IL-6, IL-8, IL-33, and HMGB1 proteins. Data are expressed as mean ± SD (*n* = 3). * *p* < 0.05 versus control group; ** *p* < 0.001 versus control group; ^#^ *p* < 0.05 versus corresponding IL-33-stimulated group; ^##^ *p* < 0.001 versus corresponding IL-33-stimulated group.

Based on the abundant expression of IL-33 cytokines in the kidney tissue of MLR/lpr mice, we speculated that IL-33 may be involved in the development of LN. To verify the ameliorating effect of quercetin on lupus nephritis phenotype, we first used recombinant human IL-33 to induce fibrosis and inflammation in HK-2 renal tubular epithelial cells. The results of immunofluorescence staining, Western blotting, and qPCR analysis showed that 50 and 100 ng/mL IL-33 significantly upregulated the expression level of fibrosis-related indicators vimentin (Figure 2A,C,D,F) and fibronectin (Figure 2B,C,E,G), whereas quercetin significantly reduced the expression of vimentin and fibronectin induced by IL-33. Similarly, the expression of inflammatory markers IL-6 (Figure 3A,B), IL-8 (Figure 3A,C), IL-33 (Figure 3A,D), and HMGB1 (Figure 3A,E) were significantly stimulated by treatment with 50 and 100 ng/mL IL-33. However, quercetin reversed the expression of IL-6, IL-8, IL-33, and HMGB1 induced by IL-33, indicating that quercetin exerts anti-fibrotic and anti-inflammatory effects in the cellular model of lupus nephritis, as opposed by IL-33.

### 3.4. Quercetin Suppresses IL-33-Stimulated Inflammasome and Pyroptosis-Associated Protein in Human Renal Tubular Epithelial Cell Line

**Figure 4:**
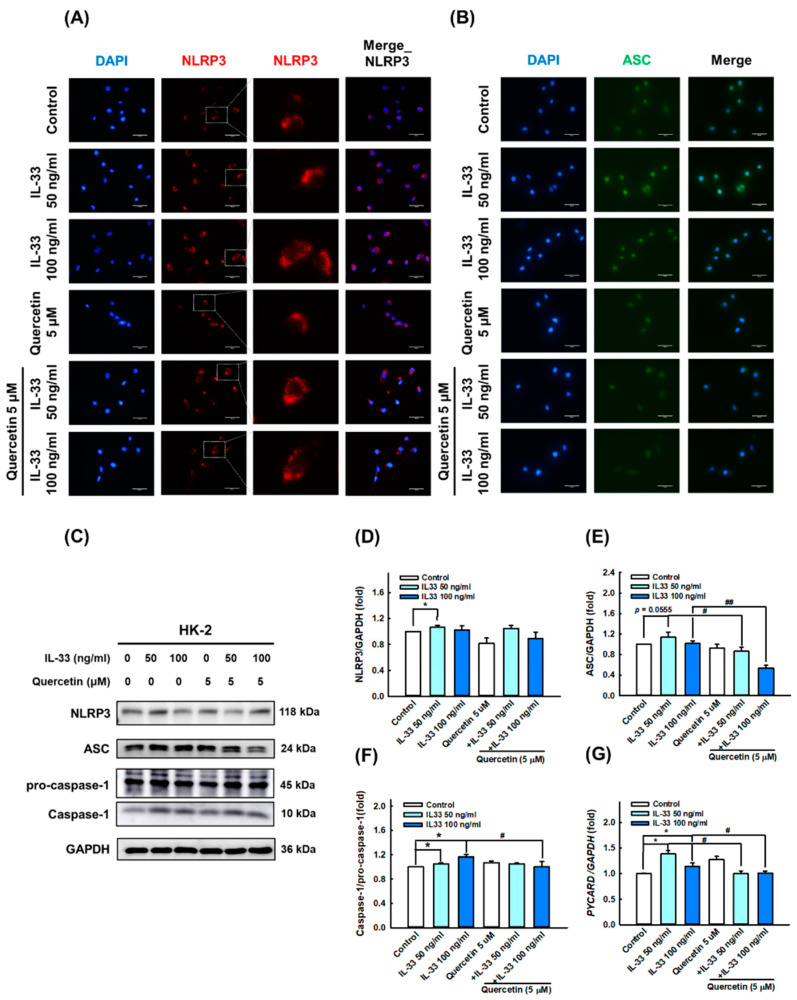
Quercetin reduced inflammasome-associated proteins expression in human renal tubular epithelial cell line. HK-2 cells were co-cultured with 5 μM quercetin and 50 ng/mL or 100 ng/mL recombinant human IL-33 for 48 h. (**A**,**B**) Representative photomicrographs showing the immunofluorescence staining for NLRP3 (red) and ASC (green) among different groups. Boxed areas are enlarged and presented in the right column. Magnification, 400×. (**C**–**F**) Immunoblots and quantitative histograms showing the results of Western blotting analysis for the expression of NLRP3, ASC, and caspase-1 proteins. (**G**) Quantitative histograms showing the results of qPCR analysis for *PYCARD* mRNA level. Data are expressed as mean ± SD (*n* = 3). * *p* < 0.05 versus control group; ^#^ *p* < 0.05 versus corresponding IL-33-stimulated group; ^##^ *p* < 0.001 versus corresponding IL-33-stimulated group.

**Figure 5:**
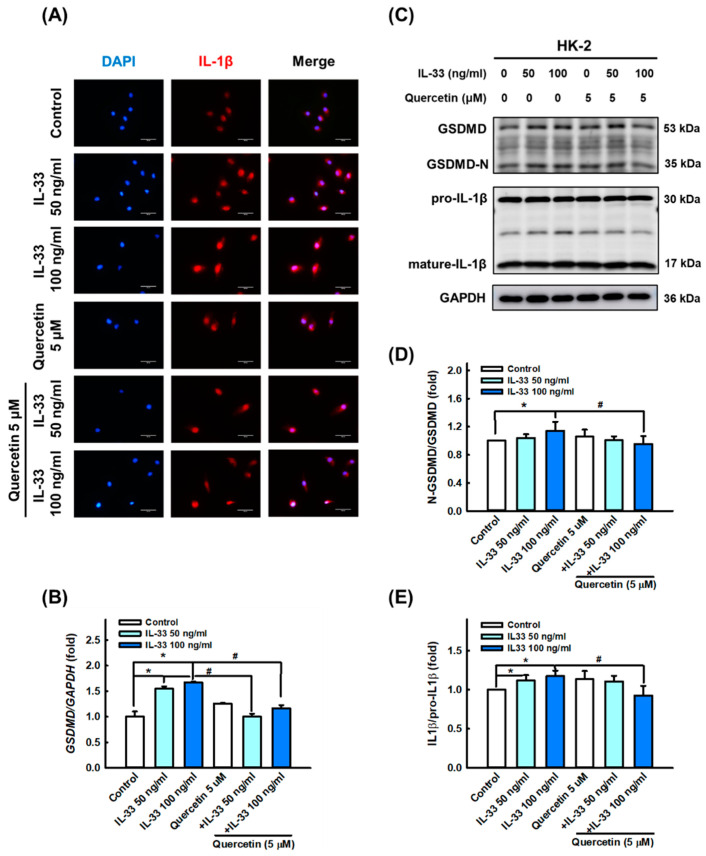
Quercetin reduced pyroptosis-associated proteins expression in human renal tubular epithelial cell line. HK-2 cells were co-cultured with 5 μM quercetin and 50 ng/mL or 100 ng/mL recombinant human IL-33 for 48 h. (**A**) Representative photomicrographs showing the immunofluorescence staining for IL-1β (red) among different groups. Magnification, 400×. (**B**) Quantitative histograms showing the results of qPCR analysis for *GSDMD* mRNA level. (**C**–**E**) Immunoblots and quantitative histograms showing the results of Western blotting analysis for the expression of GSDMD and IL-1β protein. Data are expressed as mean ± SD (*n* = 3). * *p* < 0.05 versus control group; ^#^ *p* < 0.05 versus corresponding IL-33-stimulated group.

Furthermore, we presumed that IL-33 can induce systemic inflammatory responses and damage by triggering pyroptosis in HK-2 cells, as canonical inflammasome and GSDMD activation are considered key factors in LN pathogenesis [36]. The results of immunofluorescence staining, Western blotting, and qPCR showed that IL-33 markedly increased the activation of NLRP3 (Figure 4A,C,D), ASC (Figure 4B,C,E,G), and caspase-1 (Figure 4C,F); IL-33 also enhanced the level of N-GSDMD (Figure 5B,C,D) and IL-1β secretion (Figure 5A,C,E), especially at a dose of 100 ng/mL. However, quercetin reversed the expression of NLRP3, ASC, caspase-1, N-GSDMD, and IL-1β induced by IL-33, indicating that quercetin can relieve inflammasome- and GSDMD-mediated pyroptosis in the cellular model of lupus nephritis.

### 3.5. Quercetin Regulates IL-33-Stimulated Inflammatory Pathways in Human Renal Tubular Epithelial Cell Line

**Figure 6:**
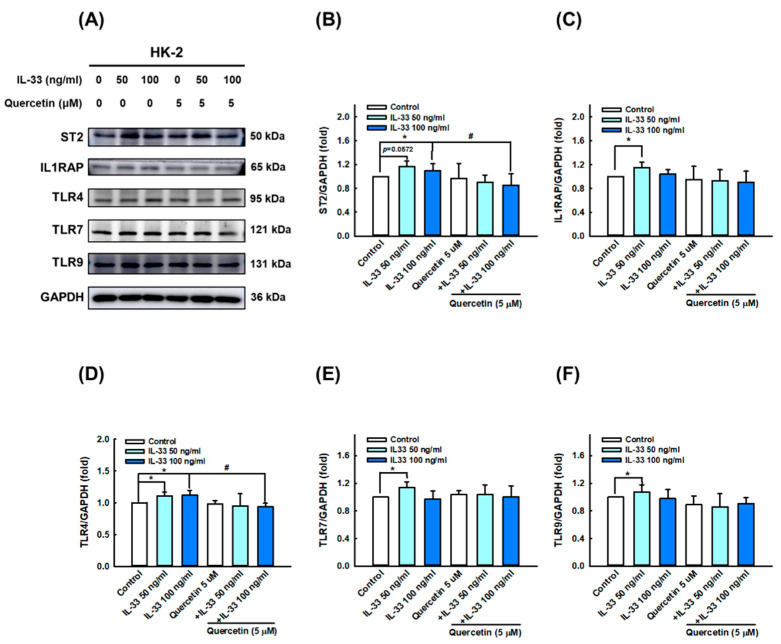
Quercetin suppressed the expression of receptor proteins in human renal tubular epithelial cell line. HK-2 cells were co-cultured with 5 μM quercetin and 50 ng/mL or 100 ng/mL recombinant human IL-33 for 48 h. (**A**–**F**) Immunoblots and quantitative histograms showing the results of Western blotting analysis for the expression of ST2, IL1RAP, TLR4, TLR7, and TLR9 proteins. Data are expressed as mean ± SD (*n* = 3). * *p* < 0.05 versus control group; ^#^ *p* < 0.05 versus corresponding IL-33-stimulated group.

**Figure 7:**
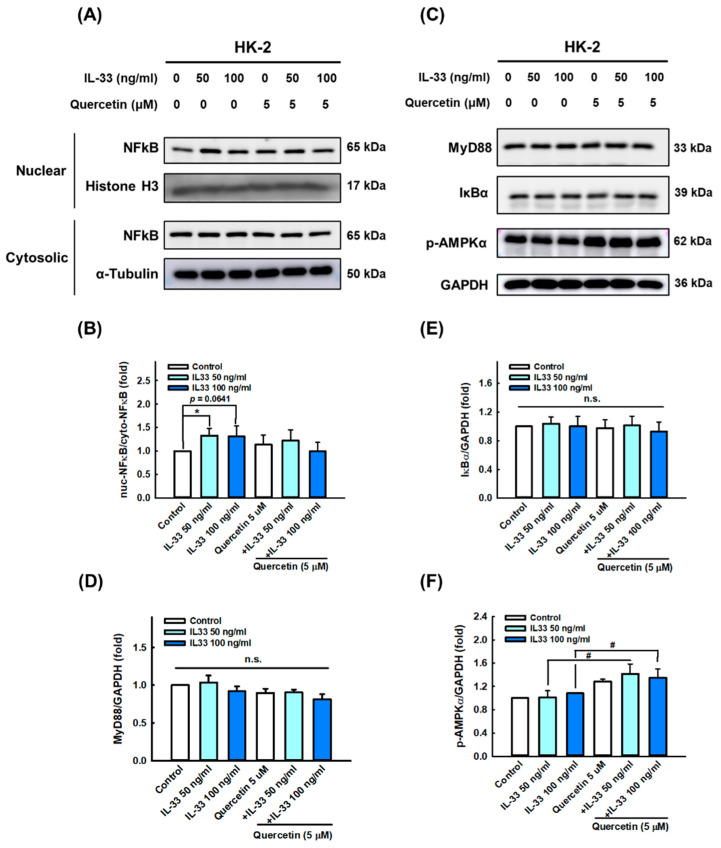
Quercetin regulated the expression of inflammation-related proteins in human renal tubular epithelial cell line. HK-2 cells were co-cultured with 5 μM quercetin and 50 ng/mL or 100 ng/mL recombinant human IL-33 for 48 h. (**A**–**F**) Immunoblots and quantitative histograms showing the results of Western blotting analysis for the expression of NF-κB, MyD88, IκBα, and p-AMPK proteins. Data are expressed as mean ± SD (*n* = 3). * *p* < 0.05 versus control group; ^#^ *p* < 0.05 versus corresponding IL-33-stimulated group; n.s. indicates not significant.

To further clarify the mechanism of quercetin effects in HK-2 cells’ differing IL-33, we first verified the expression of various receptors, including ST2 (Figure 6A,B), IL1RAP (Figure 6A,C), TLR4 (Figure 6A,D), TLR7 (Figure 6A,E), and TLR9 (Figure 6A,F) using immunoblotting. IL-33 significantly increased the activation of ST2, IL1RAP, TLR4, TLR7, and TLR9, whereas quercetin was more effective in reducing the expression of ST2 and TLR4 (Figure 6A,B,D). The expression of NF-κB, the most important transcription factor in the inflammatory pathway, was stimulated in response to IL-33, and it decreased following quercetin combination. However, its expression of downregulation was after quercetin intervention did not reach statistical significance due to a large standard deviation (Figure 7A,B). Likewise, the protein expression levels of MyD88 (Figure 7C,D) and IκBα (Figure 7C,E), as downstream targets, were not statistically different between groups. On the other hand, concerning AMPK, which is an important molecule involved in several anti-inflammatory pathways, our results showed that quercetin significantly increased p-AMPK activation, even though exposure to IL-33 did not significantly reduce p-AMPK expression (Figure 7C,F). To sum up, these results show that quercetin ameliorates inflammasome- and GSDMD-mediated pyroptosis, primarily through the regulation of IL-33/ST2 and possibly partly through the IL-33/TLR4 pathway.

## 4. Discussion

**Figure 8:**
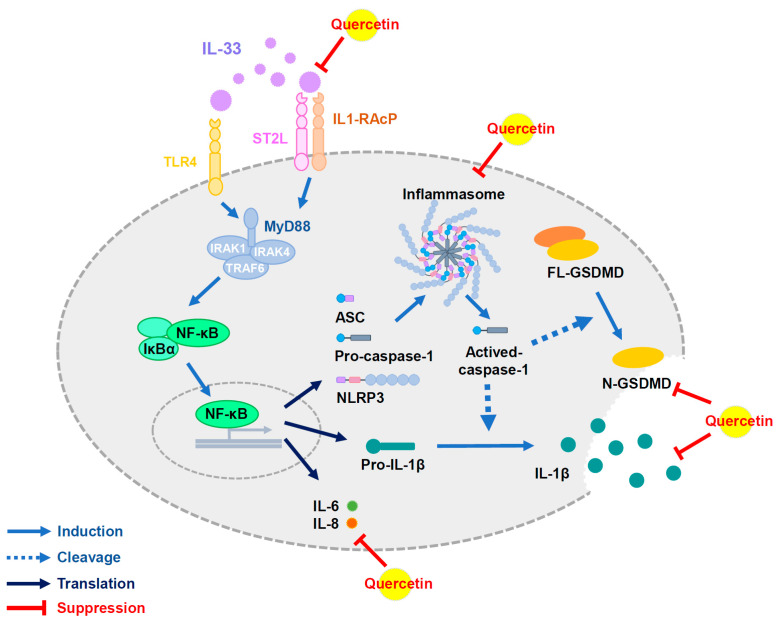
Schematic representation of the mechanisms through which quercetin exerts its potential effects in lupus nephritis-induced renal injury progression. Quercetin suppresses IL-33/ST2 signaling pathway, inhibits inflammasome and pyroptosis-related proteins expression, and reduces the secretion of IL-6 and IL-8 cytokines. Abbreviations: IL-33, interleukin-33; ST2, suppression of tumorigenicity-2; TLR4, toll-like receptors 4; IL-1RAcP, IL-1 receptor accessory protein; MyD88, myeloid differentiation primary response protein 88; IRAK, IL-1R-associated kinase; TRAF6, tumor necrosis factor receptor-associated factor 6; IκBα, inhibitor of nuclear factor κBα; NF-κB, nuclear factor-kappa B; GSDMD, gasdermin D; ASC, apoptosis-associated speck-like protein containing a caspase recruitment domain; NLRP3, NOD-like receptor family pyrin domain-containing protein 3; caspase-1, cysteine aspartate-specific proteases-1.

Lupus nephritis is one of the most common complications in patients with systemic lupus erythematosus; this study investigated whether quercetin has the potential to improve lupus nephritis in vivo and in vitro. Notably, quercetin decreased the plasma levels of BUN and creatinine, as well as ameliorating the degree of pathological progression in the kidney tissue sections of MRL/lpr mice as in in vivo models of SLE. In addition, quercetin reversed IL-33-induced fibrosis, inflammasome, and pyroptosis-related proteins expression while improving the molecular expression of inflammation-related pathways in HK-2 cells in vitro (Figure 8). The results of this study confirmed that quercetin has the potential to improve lupus nephritis.

Mice have the advantages of a shortened lifespan, ease of reproduction, ease of genetic manipulation, and the fact that standardized conditions can be maintained [37]. Spontaneous and induced models of lupus are useful tools for the study of the disease etiology. The classic models of spontaneous lupus include the NZB/W F1, MRL/lpr, and BXSB/Yaa strains, whereas induced models include the pristane-induced model and the chronic graft-versus-host-disease models (cGVHD) [38].

Studies have pointed out that several cytokines, chemokines, or growth factors have been utilized to determine the clinical pathogenesis of SLE patients [8], including interleukin subfamily, CC chemokine ligand (CCL) subfamily, transforming growth factor-β (TGFβ), etc. [39]. It is worth noting that serum IL-33 levels were found to be upregulated in SLE patients, suggesting that IL-33 may serve as a biomarker and therapeutic target for SLE [17]. A recent piece of research indicated that neutrophils from SLE patients release neutrophil extracellular traps (NETs) complexed with IL-33 in the blood and other inflamed tissues, such as kidneys and skin, and contribute to the production of type I IFNs through interactions with the ST2L receptor on plasmacytoid dendritic cells (pDCs) [40], implying that IL-33 and its receptor ST2 may contribute to the pathogenesis of autoimmune diseases, especially in patients with SLE. Moreover, Li et al. (2014) indicated that MRL/lpr mice receiving the anti-IL-33 antibody showed significantly less severe proteinuria than untreated control; moreover, serum creatinine was significantly reduced by the anti-IL-33 Ab treatment [19]. Taken together, these studies highlight the significant role of IL-33 in the development of SLE.

Tubulointerstitial fibrosis is a common defect in the progression of lupus nephritis to end-stage renal disease (ESRD) [7]. Proinflammatory cytokines or chemokines stimulate the epithelial–mesenchymal transition (EMT) pathway, which converts epithelial cells into fibroblasts, which, in turn, causes renal fibrosis [41]. Recent studies provide evidence that tubular epithelial cells may contribute to renal fibrogenesis through partial epithelial–mesenchymal transition [42]. IL-33 is not only a fundamental cytokine in inflammatory diseases but also a profibrotic protein [43]. In a previous study, IL-33 could induce EMT in HK-2 cells by promoting fibronectin expression [44]. This was consistent with our results where IL-33 significantly increased the expression of vimentin and fibronectin and stimulated the production of proinflammatory cytokines IL-6, IL-8, and HMGB1 proteins.

A previous study showed that 100 mg/kg quercetin attenuated renal interstitial fibrosis in renal tissues of UUO rats, while 40 μM quercetin inhibited the EMT and extracellular matrix (ECM) accumulation in TGF-β1-treated NRK-52E cells [45]. Our study results were consistent as collagen deposition in kidney tissue was reduced after quercetin administration, and HK-2 renal tubular epithelial cells had lower expression of vimentin and fibronectin after quercetin intervention, indicating renal fibrotic alteration in MRL/lrp mice and IL-33-induced renal tubular epithelial cell fibrosis were both improved after quercetin treatment.

Many studies have shown that quercetin and its metabolites can inhibit the production of inflammatory cytokines IL-6, IL-8, and tumor necrosis factor (TNF-α) [20]. In addition, a recent study showed that quercetin was able to reduce the inflammatory response of cells in the contrast-induced acute kidney injury (CI-AKI) model via downregulation of HMGB1 protein expression [46]. Similarly, we observed consistent results that IL-33-induced proinflammatory cytokines IL-6, IL-8, and HMGB1 in HK-2 cells were ameliorated following quercetin treatment. Thus, the secretion of pro-fibrotic proteins and cytokines, triggered in response to IL-33, were significantly reduced when quercetin was used as a treatment. These findings highlight the benefits of quercetin as a potential treatment for LN patients.

Accumulating evidence supports the involvement of the NOD-like receptor family pyrin domain-containing protein 3 (NLRP3) inflammasome in driving renal fibrosis and inflammatory responses [47,48]. The activation of the NLRP3 inflammasome promotes the activation of inflammatory caspases (caspase-1)-dependent pro-inflammatory hormones IL-1β and IL-18, which, in turn, promotes renal fibrosis [49]. Our study showed that the positive staining ratio of NLRP3 in renal tissue was decreased after quercetin administration, and the expression of NLRP3, apoptosis-associated speck-like protein containing a caspase recruitment domain (ASC) and caspase-1 in renal epithelial cells was induced by IL-33 and decreased after quercetin intervention, suggesting that renal inflammation in MRL/lrp mice and IL-33-induced inflammation-related molecules in HK-2 cells were both ameliorated after quercetin treatment.

Pyroptosis is a type of lytic cell death triggered by inflammatory caspases that are normally activated in multiprotein inflammasome complexes assembled in response to pathogens and endogenous danger signals [50]. Notably, gasdermin D (GSDMD) was identified as a caspase substrate and considered an important mediator of pyroptosis in processes involving cleavage by active caspase-1 within the junction of the N- and C-terminal domains of GSDMD. Afterward, N-GSDMD promotes the formation of the plasma membrane pore [51], as well as stimulating pyroptosis with a subsequent production of cytokines IL-1β and IL-18 [52]. Our study showed that the expression of N-GSDMD and IL-1β in renal epithelial cells was induced by IL-33 and decreased after quercetin intervention, concluding that IL-33-induced pyroptosis-related molecules in HK-2 cells were ameliorated after quercetin treatment.

The canonical pathway elicited by IL-33 results in stimulation of a receptor complex composed of ST2L and interleukin 1 receptor accessory protein (IL-1RAP) [43], which triggers the recruitment of MyD88, IRAK1, IRAK4, and TRAF6, followed by activation of downstream signaling pathways, such as NF-κB, c-Jun N-terminal kinase (JNK), p38, and ERK [53]. A previous study in STZ-induced diabetic rats found that quercetin intervention downregulated the expression of NF-κB in the kidney [54]. Similarly, our current data showed that IL-33 significantly induced ST2, IL-1RAP, and NF-κB expression, whereas quercetin only reduced ST2 expression, indicating that quercetin can affect the inflammatory status of renal tubular epithelial cells by inhibiting ST2 receptors. However, the changes of downstream mechanisms still need to be further explored.

Toll-like receptors (TLRs) are pattern recognition receptors (PRRs) that play an important role in innate immunity and are typically expressed on macrophages, dendritic cells, and B lymphocytes, and/or epithelial and mesangial cells of the kidney [55]. In the pathogenesis of SLE, TLRs are activated by exogenous ligands of infection and/or endogenous ligands of apoptotic debris that are not cleared, and downstream signaling cascades are initiated, leading to the production of inflammatory cytokines [55]. A recent study by Upadhyay et al. found that the knockdown of TLR9 and reactive oxygen species (ROS) inhibition ameliorated EMT, mitochondrial dysfunction, and inflammatory response in proximal renal tubular cells induced by aristolochic acids [56]. In the present study, our findings showed that IL-33 significantly induced the expression of TLR4, TLR7, and TLR9 proteins, whereas quercetin only reduced IL-33-induced TLR4 expression, suggesting that quercetin may alter the release of inflammation-related proteins through TLR4, located on the cell surface, rather than TLR7 and TLR9 on the membranes of endosomes.

AMP-activated protein kinase (AMPK), a heterotrimeric serine/threonine protein kinase, is an important regulator of whole-body energy metabolism [57]. It is well known that activation of AMPK contributes to the prevention of inflammation and NF-κB pathway transactivation [58]. A concurrent study showed that quercetin attenuated oxidative stress-mediated endoplasmic reticulum (ER) stress and mitochondrial dysfunction through activating SIRT1/AMPK signal pathways in lipopolysaccharide (LPS)-stimulated murine lung epithelial (MLE-12) cells [59]. Consistent with our observations, quercetin significantly induced p-AMPKα expression in HK-2 cells even under IL-33 induction, indicating an anti-inflammatory effect of quercetin. On the other hand, previous literature showed that IL-33 induced an increase in p-AMPK expression in group 2 innate lymphoid cells (ILC2) in a short period of time, but the negative feedback would decrease p-AMPK expression due to the prolonged induction of IL-33 [60]. In addition, IL-33 induced p-AMPK expression in monocytes to promote mitophagy [61]. However, in our experiment, the intervention of IL-33 did not significantly change the expression of p-AMPK. We speculate that it may be closely related to the time of intervention, which needs more experiments to confirm.

There are some limitations to be aware of in this experiment. First, quercetin is considered a potent and well-known antioxidant [21], however, our present results found that IL-33-induced renal epithelial cell injury does not appear to occur through the promotion of oxidative stress (shown in Supplementary Figure S2). Furthermore, after searching the database with the same criteria, it was found that IL-33 was less explored in renal epithelial cells and oxidative stress studies. Therefore, the antioxidant capacity of quercetin does not apply to this conclusion after being challenged with IL-33. Second, the results indicated that changes in AMPKα were not the main factor of IL-33-induced inflammation and fibrosis, although quercetin induced a significant increase in AMPKα expression. Therefore, we speculate that other mechanisms may be involved in the anti-inflammatory or anti-fibrotic effects of quercetin. However, the exact pathway requires more experimental confirmation. Finally, many studies remain skeptical about the role of IL-33 in SLE patients, and two opposing views have emerged. Fortunately, this study proposes novel findings. We used IL-33 to induce renal proximal tubule epithelial cells to mimic the fibrosis and inflammatory responses that LN may encounter, which was then treated with quercetin. This positive impact indicates that quercetin can indeed improve the renal fibrosis by retarding the inflammatory response in LN development.

## 5. Conclusions

In summary, our findings showed that quercetin was able to decrease the plasma levels of BUN and creatinine and ameliorate the degree of pathological deterioration in kidney tissue sections in MRL/lpr mice in an in vivo SLE animal model. Furthermore, we reported for the first time that quercetin reversed IL-33-induced fibrosis, inflammasome, and pyroptosis-related protein expression in HK-2 cells via IL-33/ST2 in vitro. Collectively, these findings propose quercetin as a potential therapeutic option in the management of lupus nephritis.

## Data Availability

Data are contained within the article or Supplementary material.

## Supplementary Materials

The following supporting information can be downloaded at https://www.mdpi.com/article/10.3390/antiox11112238/s1, Figure S1: Quercetin ameliorates unilateral ureteral obstruction (UUO)-induced renal injury, Supplementary Figure S2: Effects of quercetin and IL-33 on reactive oxygen species (ROS) production in HK-2 human renal tubular epithelial cells; Supplementary Table S1: The detailed information about used antibodies, Supplementary Table S2: The detailed information about used primers.
