# Supplementary material for: Quercetin Ameliorates Renal Injury and Pyroptosis in Lupus Nephritis through Inhibiting IL-33/ST2 Pathway In Vitro and In Vivo
Source: Antioxidants (Basel). 2022 Nov 13;11(11):2238. doi: 10.3390/antiox11112238 (PMC9687047; doi:10.3390/antiox11112238)
Supplement: Supplementary file 1 [file antioxidants-11-02238-s001.zip › antioxidants-1998804-supplementary.pdf]

Quercetin ameliorates renal injury and pyroptosis in lupus nephritis through inhibiting IL-33/ST2 pathway *In vitro* and *In vivo*

-- **Supplementary figures**

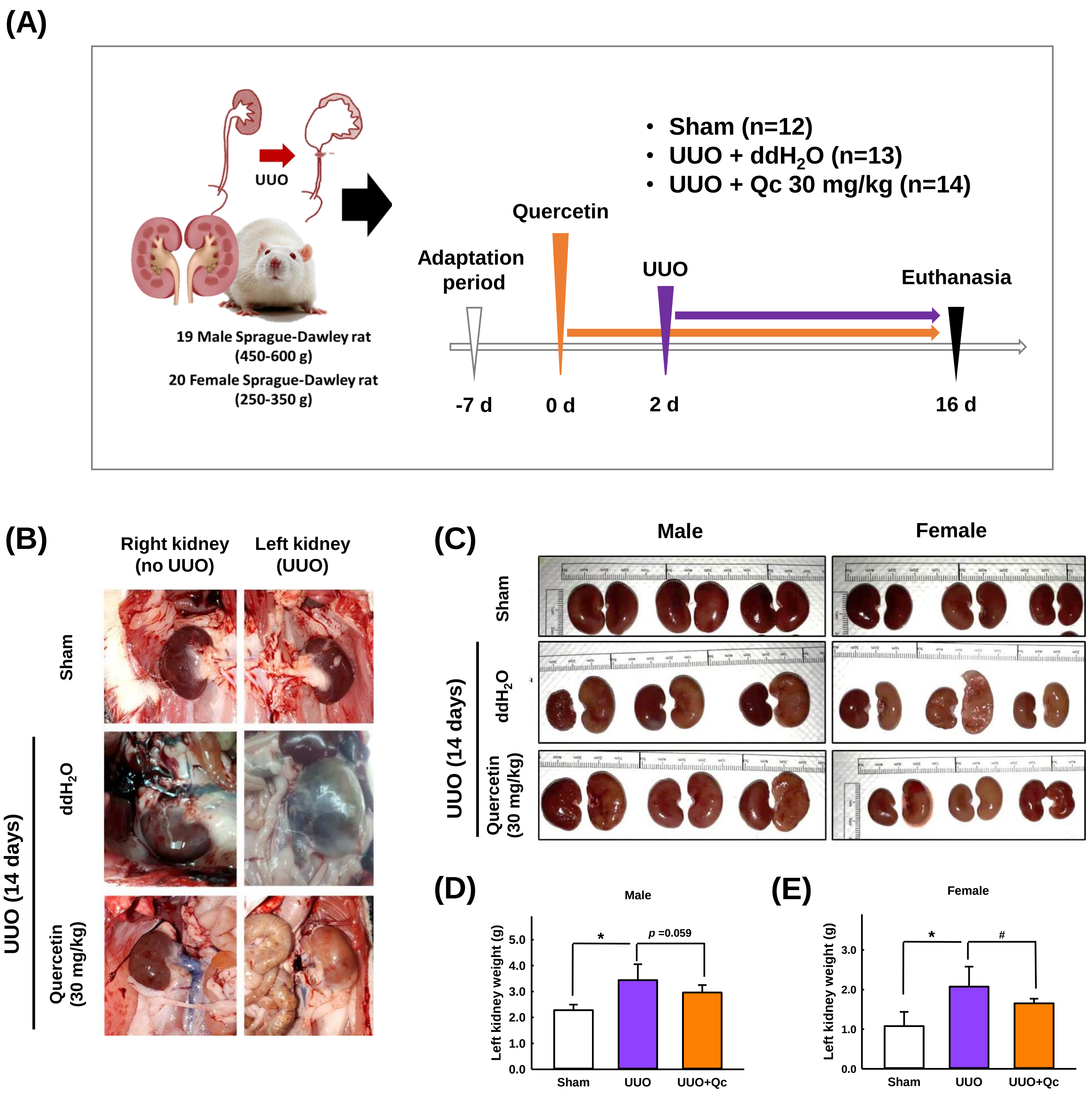

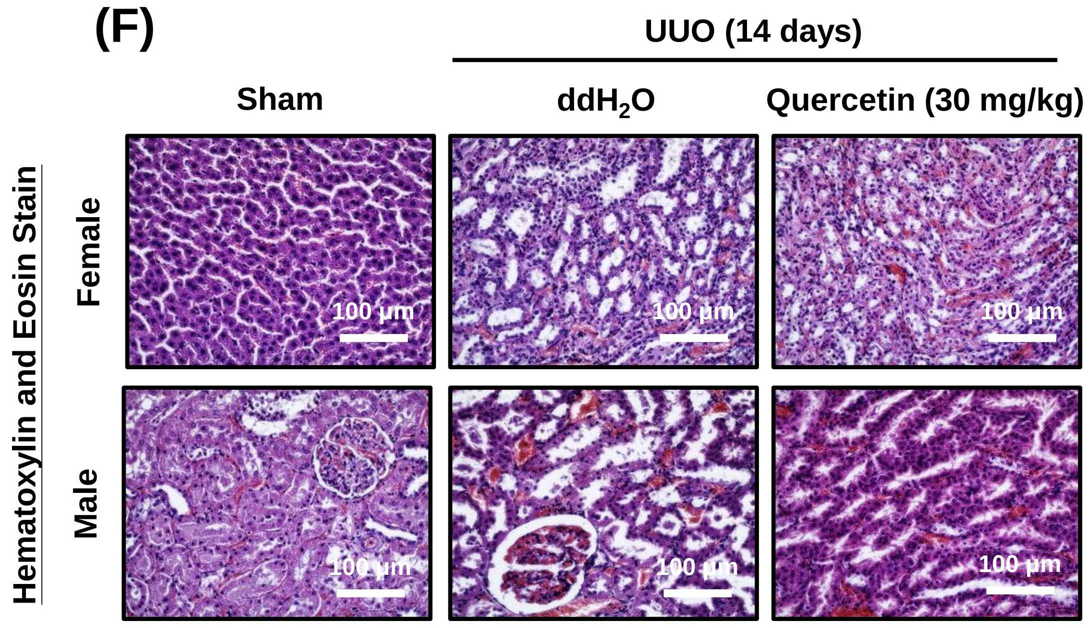

(G)

Serum biomarker	CF	UF	QF
Kidney function biomarkers			
BUN (mg/ dL)	16 ± 2.2	43 ± 5.8 *	25 ± 2.6 #
Creatinine (mg/ dL)	0.55 ± 0.1	2.1 ± 0.7 *	0.8 ± 0.2 #
**Serum biomarker**	**CM**	**UM**	**QM**
Kidney function biomarkers			
BUN (mg/ dL)	16 ± 3.8	39 ± 4.2 *	22 ± 2.6 #
Creatinine (mg/ dL)	0.5 ± 0.2	1.9 ± 0.8 *	0.9 ± 0.3 #

Supplementary Figure S1. Quercetin ameliorates unilateral ureteral obstruction (UUO)-induced renal injury. (A) *In vivo* experimental procedure. (B—E) Changes in appearance and weight of kidney in UUO rat. (F) Renal tissue specimen stained with hematoxylin and eosin stain for UUO rat that were given either vehicle (ddH_2O) or quercetin (30 mg/kg). Magnification, x40. G) Determination of blood urea nitrogen (BUN) and plasma creatinine concentration. Data were expressed as mean ± SD for each group. *p< 0.05 versus sham control; #p< 0.05 versus UUO group.

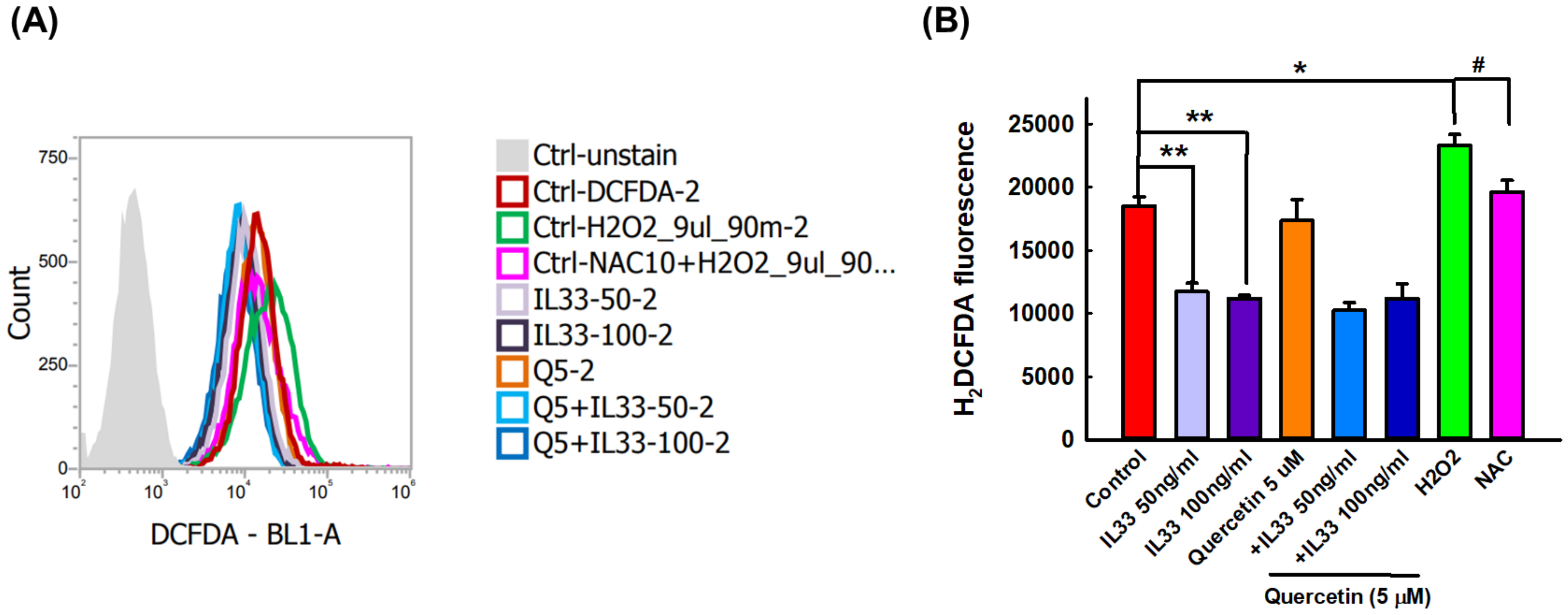

Supplementary Figure S2. Effects of quercetin and IL-33 on reactive oxygen species (ROS) production in HK-2 human renal tubular epithelial cells. (A) Flow cytometry results of H_2-DCFDA staining of HK-2 cells treated with different interventions. (B) Quantitative histogram after treatment with different interventions. Data were expressed as mean ± SD for each group. **p*< 0.05 or ***p*< 0.001 versus control; $^{\#}p < 0.05$ versus H_2O_2 group. Abbreviations: IL-33, interleukin-33; H_2O_2, hydrogen peroxide; H_2-DCFDA, 2',7'-dichlorodihydrofluorescein diacetate; NAC, N-acetylcysteine; Q, quercetin.

Supplementary Table S1. The detailed information about used antibodies.

**Antibody**	**Source**	**Catalogue number**	**Dilution rate**
IL-33	Affinity Biosciences	DF8319	IHC: 1:400<br>WB: 1:1000
GSDMD	Affinity Biosciences	AF4012	WB: 1:1000
Fibronectin	Abcam	Ab2413	ICC: 1:200<br>WB: 1:1000
Histone H3	Abcam	Ab1791	WB: 1:1000
α-Tubulin	Abcam	Ab7291	WB: 1:10000
IκBα	Cell Signaling Technology	#4814	WB: 1:1000
MyD88	Cell Signaling Technology	#4283	WB: 1:1000
p-AMPKα	Cell Signaling Technology	#2535	WB: 1:1000
NF-κB	Cell Signaling Technology	#4764	WB: 1:1000
Vimentin	GeneTex	GTX100619	ICC: 1:200<br>WB: 1:15000
TLR9	GeneTex	GTX100726	WB: 1:1000
IL1RAP	GeneTex	GTX104513	WB: 1:1000
NLRP3	Novus Biologicals	NBP2-12446	IHC: 1:400<br>ICC: 1:200<br>WB: 1:1000
IL-6	Novus Biologicals	NB600-1131	WB: 1:1000
HMGB-1	Novus Biologicals	NB100-2322	WB: 1:1000
IL-1β	Proteintech	Ab9722	IHC: 1:400<br>ICC: 1:200<br>WB: 1:1000
IL-8	Proteintech	27095-1-AP	WB: 1:1000
ST2	Proteintech	11920-1-AP	WB: 1:1000
TLR7	Proteintech	17232-1-AP	WB: 1:1000
GAPDH	Proteintech	60004-1-Ig	WB: 1:10000
ASC	Santa Cruz Biotechnology	sc-271054	ICC: 1:200<br>WB: 1:500
caspase-1	Santa Cruz Biotechnology	sc-56036	WB: 1:500
TLR4	Santa Cruz Biotechnology	sc-293072	WB: 1:800

Supplementary Table S2. The detailed information about used primers.

Accession number	Gene	Forward (5′ to 3′)	Reverse (5′ to 3′)
NM_003380.5	*VIM*	AGTCCACTGAGTACCGGAGAC	CATTTCACGCATCTGGCGTTC
NM_002026.4	*FN1*	CGAGCTTCCCCAACTGGTAACCC	AGCTTCTTGTCCTACATTCGGCGG
NM_145182.3	*PYCARD*	TGGATGCTCTGTACGGGAAG	CCAGGCTGGTGTGAAACTGAA
NM_024736.7	*GSDMD*	GGACAGGCAAAGATCGCAG	CACTCAGCGAGTACACATTCATT
NM_001357943.2	*GAPDH*	TGCACCACCAACTGCTTAGC	GGCATGGACTGTGGTCATGAG
